# Sequence of inequalities among fuzzy mean difference divergence measures and their applications

**DOI:** 10.1186/2193-1801-3-623

**Published:** 2014-10-22

**Authors:** Vijay Prakash Tomar, Anshu Ohlan

**Affiliations:** Department of Mathematics, Deenbandhu Chhotu Ram University of Science & Technology, Murthal, 131039 Haryana India

**Keywords:** Pattern recognition, Fuzzy entropy, Fuzzy divergence measure, Inequalities, Fuzzy mean difference divergence measures

## Abstract

This paper presents a sequence of fuzzy mean difference divergence measures. The validity of these fuzzy mean difference divergence measures is proved axiomatically. In addition, it introduces a sequence of inequalities among some of these fuzzy mean difference divergence measures. The applications of proposed fuzzy mean difference divergence measures in the context of pattern recognition have been presented using a numerical example. It is shown that the proposed fuzzy mean difference divergence measures are well suited to use with linguistic variables. Finally, on establishing inequalities, we find that our proposed measures are computationally much more efficient.

## Introduction

Shannon (1948) was first to use the word “entropy” to measure an uncertain degree of the randomness in a probability distribution. Entropy as a measure of fuzziness was first introduced by Zadeh (1968). There is an intrinsic similarity between two equations however Shannon entropy measures the average uncertainty in bits associated with the prediction of outcomes in a random experiment, but the entropy of fuzzy set describes the degree of fuzziness in a fuzzy set. The concept of fuzzy sets proposed by Zadeh (1968) has proven useful in the context of pattern recognition, image processing, speech recognition, bioinformatics, fuzzy aircraft control, feature selection, decision making, etc.

Entropy, as a very important notion for measuring fuzziness degree or uncertain information in fuzzy set theory, has received a great attention. For example, Kullback and Leibler (1951) obtained the measure of directed divergence between two probability distributions. Bhandari and Pal (1993) presented some axioms to describe the measure of directed divergence between fuzzy sets, which is proposed corresponding to Kullback and Leibler (1951) measure of directed divergence. Thereafter, many other researchers have studied the fuzzy divergence measures in different ways and provide their application in different areas. In 1999, Fan and Xie introduced the divergence measure based on exponential operation and studied its relation with divergence measure introduced in Bhandari and Pal (1993). Montes et al. (2002) studied the special classes of divergence measures and used the link between fuzzy and probabilistic uncertainty. Parkash et al. (2006) proposed two fuzzy divergence measures corresponding to Ferreri (1980) probabilistic measure of directed divergence. Ghosh et al. (2010) gave the application of Bhandari and Pal (1993) divergence measure in the area of automated leukocyte recognition. Bhatia and Singh (2012) proposed the fuzzy divergence measure corresponding to Taneja (2008) Arithmetic–geometric divergence measure.

In the recent years, many authors have introduced various divergence measures between fuzzy sets. We introduce a sequence of fuzzy mean difference divergence measures and established the inequalities among them to explore the fuzzy inequalities. The advantage of establishing the inequalities is to make the computational work much simpler. The technique of inequalities provides a better comparison among fuzzy mean divergence measures.

### Preliminaries on fuzzy divergence measures

Fuzziness, a feature of uncertainty, results from the lack of sharp difference of being or not being a member of the set, i.e., the boundaries of the set under consideration are not sharply defined. A fuzzy set *A* defined on a universe of discourse *X* is given as Zadeh (1965):


where μ_*A*_ : *X* → [0, 1] is the membership function of *A*. The membership value μ_*A*_(*x*) describes the degree of the belongingness of *x* ∈ *X* in *A*. When μ_*A*_(*x*) is valued in {0, 1}, it is the characteristic function of a crisp (non-fuzzy) set. Zadeh (1965) gave some notions related to fuzzy sets, one of them which we shall need in our discussion, is as follows:
1

The measure of information defined by Shannon (1948) is given by
2

Taking into consideration the concept of fuzzy sets, De Luca and Termini (1972) introduced the measure of fuzzy entropy corresponding to Shannon’s entropy given in (2) as
3

Kullback and Leibler (1951) obtained the measure of directed divergence of probability distribution *P* = (*p*_1_, *p*_2_, … *p*_*n*_) from probability distribution *Q* = (*q*_1_, *q*_2_, … *q*_*n*_) as
4

Measure of fuzzy divergence between two fuzzy sets gives the difference between two fuzzy sets and this measure of distance/difference between two fuzzy sets is called the fuzzy divergence measure.

Bhandari and Pal (1993) introduced the measure of fuzzy directed divergence corresponding to (4) as
5

The fuzzy mean divergence measures corresponding to seven geometrical mean measures given in Taneja (2012) are presented in Table 1.

**Table 1:** **Fuzzy mean divergence measures**

Sr. no.	Fuzzy mean divergence measure	Definition
1.	Fuzzy Arithmetic Mean Measure	
2.	Fuzzy Geometric Mean Measure	
3.	Fuzzy Harmonic Mean Measure	
4.	Fuzzy Heronian Mean Measure	
5.	Fuzzy Contra-harmonic Mean Measure	
6.	Fuzzy Root-mean-square Mean Measure	
7.	Fuzzy Centroidal Mean Measure	

We have the following Lemma in fuzzy context corresponding to the Lemma of Taneja (2005):

**Lemma 1:** Let *f* : *I* ⊂ *R*_+_ → *R* be a convex and differentiable function satisfying . Consider a function


Then the function *φ*_*f*_(*a*, *b*) is convex . Additionally, if *f*′(1/2) = 0, then the following inequality holds:


**Lemma 2: Schwarz’s Lemma:** Let *f*_1_, *f*_2_ : *I* ⊂ *R*_+_ → *R* be two convex functions satisfying the assumptions:

i);
ii) *f*_1_ and *f*_2_ are twice differentiable in *R*_+_;
iii) there exist the real constants *α*, *β* such that 0 ≤ *α* < *β* and , , for all *z* > 0 then we have the inequalities:for all *a*, *b* ∈ (0, 1), where the function *φ*_(.)_(*a*, *b*) is defined as

## Results and discussion

### Sequence of fuzzy mean difference divergence measures

Corresponding to the fuzzy mean divergence measures defined in Table 1, we propose a sequence of fuzzy mean difference divergence measures as follows:
67891011121314151617181920212223

**Theorem 1:** All the proposed measures (6) - (23) are valid measures of fuzzy mean difference directed divergence.

**Proof: (a) Non-negativity:** From one of inequality in Taneja (2012), for two fuzzy sets *A* and *B*, we have *H*(*A*, *B*) ≤ *G*(*A*, *B*) ≤ *N*(*A*, *B*) ≤ *A*(*A*, *B*) ≤ *R*(*A*, *B*) ≤ *S*(*A*, *B*) ≤ *C*(*A*, *B*).

Hence, the condition of non-negativity of measures (6) - (23) is proved.

Also clearly,  for all measures from (6) - (23) where *A*_1_ and *B*_1_ belongs to the fuzzy mean divergence measures given in Table 1.

**(b) Invariant under complementation:** From the notion of compliment of a fuzzy set given in (1) we can easily check for measures (6) - (23) that .

**(c) Convexity:** Now we shall prove the condition of convexity of measures (6) - (23) with the help of Lemma 1.

For simplicity, Let us write  where  with *A*_1_ ≥ *B*_1_.

Let us take *μ*_*A*_ = *z* ⇒ *μ*_*B*_ = 1 − *z*. So, corresponding to measures (6) - (23) we have the following generating functions:
242526272829303132333435363738394041

Now in all the cases from (24) - (41), we can easily check that . It is understood that *z* ∈ [0, 1].

The first and second order derivatives of the functions (24) - (41) are as follows:
424344454647484950515253545556575859

We see that in the entire cases second order derivative are positive and satisfies  for all *z* ∈ [0, 1]. Thus according to the Lemma 1 and equation (42) - (59), we get the convexity of the measures (6) - (23).

Hence all the defined measures (6) - (23) are valid measures of fuzzy mean difference directed divergence.

### Inequalities among fuzzy mean difference divergence measures

**Theorem 2:** The fuzzy mean difference divergence measures defined in (6) - (23) admit the following inequalities:


i.e., we have the following inequalities:

i),
ii),
iii),
iv).

**Proof:** The proof of the above theorem is based on Lemma 2 and is given in parts in the following propositions.

**Proposition 1:** We have 

**Proof:** Let us consider the function


This gives


And we have
60

Applying Lemma 2 for the difference of fuzzy means *D*_*SA*_(*A*, *B*) and *D*_*SN*_(*A*, *B*) and using (60), we get


**Proposition 2:** We have 

**Proof:** Let us consider the function


This gives


And we have
61

Applying Lemma 2 for the difference of fuzzy means *D*_*SA*_(*A*, *B*) and *D*_*SH*_(*A*, *B*) and using (61), we get


**Proposition 3:** We have 

**Proof:** Let us consider the function


This gives


And we have
62

Applying Lemma 2 for the difference of fuzzy means *D*_*SH*_(*A*, *B*) and *D*_*CR*_(*A*, *B*) and using (62), we get


**Proposition 4:** We have 

**Proof:** Let us consider the function


This gives


And we have
63

Applying Lemma 2 for the difference of fuzzy means *D*_*CR*_(*A*, *B*) and *D*_*CN*_(*A*, *B*) and using (63), we get


**Proposition 5:** We have 

**Proof:** Let us consider the function


This gives


And we have
64

Applying Lemma 2 for the difference of fuzzy means *D*_*CR*_(*A*, *B*) and *D*_*SG*_(*A*, *B*) and using (64), we get


**Proposition 6:** We have 

**Proof:** Let us consider the function


This gives


And we have
65

Applying Lemma 2 for the difference of fuzzy means *D*_*SN*_(*A*, *B*) and *D*_*CN*_(*A*, *B*) and using (65), we get


**Proposition 7:** We have 

**Proof:** Let us consider the function


This gives


And we have
66

Applying Lemma 2 for the difference of fuzzy means *D*_*SN*_(*A*, *B*) and *D*_*SG*_(*A*, *B*) and using (66), we get


**Proposition 8:** We have 

**Proof:** Let us consider the function


This gives


And we have
67

Applying Lemma 2 for the difference of fuzzy means *D*_*CN*_(*A*, *B*) and *D*_*CS*_(*A*, *B*) and using (67), we get


**Proposition 9:** We have *D*_*CS*_ ≤ 3*D*_*AN*_

**Proof:** Let us consider the function


This gives


And we have
68

Applying Lemma 2 for the difference of fuzzy means *D*_*CS*_(*A*, *B*) and *D*_*AN*_(*A*, *B*) and using (68), we get


**Proposition 10:** We have 

**Proof:** Let us consider the function


This gives


And we have
69

Applying Lemma 2 for the difference of fuzzy means *D*_*CN*_(*A*, *B*) and *D*_*CG*_(*A*, *B*) and using (69), we get


**Proposition 11:** We have 

**Proof:** Let us consider the function


This gives


And we have
70

Applying Lemma 2 for the difference of fuzzy means *D*_*SG*_(*A*, *B*) and *D*_*RG*_(*A*, *B*) and using (70), we get


**Proposition 12:** We have 

**Proof:** Let us consider the function


This gives


And we have
71

Applying Lemma 2 for the difference of fuzzy means *D*_*CG*_(*A*, *B*) and *D*_*RG*_(*A*, *B*) and using (71), we get


**Proposition 13:** We have *D*_*RG*_ ≤ 5*D*_*AN*_

**Proof:** Let us consider the function


This gives


And we have
72

Applying Lemma 2 for the difference of fuzzy means *D*_*RG*_(*A*, *B*) and *D*_*AN*_(*A*, *B*) and using (72), we get


### Application of fuzzy mean difference divergence measures to pattern recognition

We now present the application of the proposed fuzzy mean difference divergence measures in the context of pattern recognition. Next, an example related to pattern recognition is given to demonstrate the results obtained by the fuzzy mean difference divergence measures (6) - (23).

In order to demonstrate the application of the introduced fuzzy mean difference divergence measures to pattern recognition, suppose that we are given three known patterns *P*_1_, *P*_2_ and *P*_3_ which have classifications *C*_1_, *C*_2_ and *C*_3_ respectively. The patterns are represented by the following fuzzy sets in the universe of discourse *X* = {*x*_1_, *x*_2_, *x*_3_, *x*_4_}:


Given an unknown pattern *Q*, represented by the fuzzy set


Our aim is to classify *Q* to one of the classes *C*_1_, *C*_2_ and *C*_3_. According to the principle of minimum divergence/discrimination information between fuzzy sets, the process of assigning *Q* to  is described by


Table 2 presents *D*_*AB*_(*P*_*k*_, *Q*), *k* = {1, 2, 3}. It is observed that Q has been classified to *C*_3_ correctly.

**Table 2:** **Computed values of fuzzy mean difference divergence measures** *D*_*AB*_(*P*_*k*_, *Q*) **with** *k* = {1, 2, 3}

	***P***_1_	***P***_2_	***P***_3_
*Q*	0.2741^(6)^	0.2877^(6)^	0.1385^(6)^
	0.5825^(7)^	0.6430^(7)^	0.3127^(7)^
	0.8002^(8)^	0.8400^(8)^	0.4064^(8)^
	0.3423^(9)^	0.3631^(9)^	0.1772^(9)^
	0.5135^(10)^	0.5446^(10)^	0.2659^(10)^
	1.0270^(11)^	1.0892^(11)^	0.5318^(11)^
	0.2385^(12)^	0.2569^(12)^	0.1274^(12)^
	0.3340^(13)^	0.3553^(13)^	0.1742^(13)^
	0.5252^(14)^	0.5523^(14)^	0.2679^(14)^
	0.7520^(15)^	0.8015^(15)^	0.3933^(15)^
	0.1712^(16)^	0.1815^(16)^	0.0887^(16)^
	0.2667^(17)^	0.2799^(17)^	0.1355^(17)^
	0.4579^(18)^	0.4769^(18)^	0.2292^(18)^
	0.6847^(19)^	0.7261^(19)^	0.3546^(19)^
	0.0955^(20)^	0.0984^(20)^	0.0468^(20)^
	0.2867^(21)^	0.2954^(21)^	0.1405^(21)^
	0.5135^(22)^	0.5446^(22)^	0.2659^(22)^
	0.1912^(23)^	0.1970^(23)^	0.0937^(23)^

For convenience, we use the notation * ^(*i*)^ in Table 2 to present the divergence/distance value computed from equation *i*.

### Numerical example

We now establish that the proposed fuzzy mean difference divergence measures (6) - (23) are reliable in applications with compound linguistic variables.

**Example:** Let *F* = {(*x*, *μ*_*F*_(*x*))/*x* ∈ *X*} be a fuzzy set in X. Tomar and Ohlan (2014) defined for any positive real number *n*, from the operation of power of a fuzzy set: *F*^*n*^ = {(*x*, [*μ*_*F*_(*x*)]^*n*^)/*x* ∈ *X*}.

Using the above operation, the concentration and dilation of a fuzzy set *F* are as follows:


CON (*F*) and DIL (*F*) are treated as “very (*F*)” and “more or less (*F*)”, respectively.

We consider *F* in *X* = {*x*_1_, *x*_2_, *x*_3_, *x*_4_, *x*_5_} defined as:


By taking into account the characterization of linguistic variables, we regard *F* as “LARGE” in X. Using the operations of concentration and dilation

*F*^1/2^ may be treated as “More or less LARGE”,

*F*^2^ may be treated as “Very LARGE”,

*F*^4^ may be treated as “Vey very Large”.

The proposed fuzzy mean difference divergence measures are used to calculate the degree of divergence/distance between these fuzzy sets. The divergence/distance values have been calculated by Eqs. (6) - (23) between different fuzzy sets. The comparative results are summarized in Table 3. For convenience, we use the notation * ^(*i*)^ in Table 3 to present the divergence/distance value computed from equation *i*. The following abbreviated notions are used in Table 3.

**Table 3:** **Divergence/distance values calculated by Eqs. (** **6** **)** - **(** **23** **)**

L.	M.L.L.	V.L.	V.V.L.	L.	M.L.L.	V.L.	V.V.L.	L.	M.L.L.	V.L.	V.V.L.	L.	M.L.L.	V.L.	V.V.L.
L.				M.L.L.				V.L.				V.V.L.			
0.0000^(6)^	0.0884^(6)^	0.1304^(6)^	0.4250^(6)^	0.0884^(6)^	0.0000^(6)^	0.4523^(6)^	0.8566^(6)^	0.1304^(6)^	0.4523^(6)^	0.0000^(6)^	0.1114^(6)^	0.4250^(6)^	0.8566^(6)^	0.1114^(6)^	0.0000^(6)^
0.0000^(7)^	0.2249^(7)^	0.2945^(7)^	0.9380^(7)^	0.2249^(7)^	0.0000^(7)^	1.0043^(7)^	1.9197^(7)^	0.2945^(7)^	1.0043^(7)^	0.0000^(7)^	0.2485^(7)^	0.9380^(7)^	1.9197^(7)^	0.2485^(7)^	0.0000^(7)^
0.0000^(8)^	0.3013^(8)^	0.3836^(8)^	1.2589^(8)^	0.3013^(8)^	0.0000^(8)^	1.3277^(8)^	2.5594^(8)^	0.3836^(8)^	1.3277^(8)^	0.0000^(8)^	0.3262^(8)^	1.2589^(8)^	2.5594^(8)^	0.3262^(8)^	0.0000^(8)^
0.0000^(9)^	0.1124^(9)^	0.1666^(9)^	0.5183^(9)^	0.1124^(9)^	0.0000^(9)^	0.5617^(9)^	0.6388^(9)^	0.1666^(9)^	0.5617^(9)^	0.0000^(9)^	0.1399^(9)^	0.5183^(9)^	0.6388^(9)^	0.1399^(9)^	0.0000^(9)^
0.0000^(10)^	0.1866^(10)^	0.2499^(10)^	0.7775^(10)^	0.1866^(10)^	0.0000^(10)^	0.8426^(10)^	1.5441^(10)^	0.2499^(10)^	0.8426^(10)^	0.0000^(10)^	0.2097^(10)^	0.7775^(10)^	1.5441^(10)^	0.2097^(10)^	0.0000^(10)^
0.0000^(11)^	0.4092^(11)^	0.4998^(11)^	1.5550^(11)^	0.4092^(11)^	0.0000^(11)^	1.6852^(11)^	3.0882^(11)^	0.4998^(11)^	1.6852^(11)^	0.0000^(11)^	0.4194^(11)^	1.5550^(11)^	3.0882^(11)^	0.4194^(11)^	0.0000^(11)^
0.0000^(12)^	0.0982^(12)^	0.1195^(12)^	0.3525^(12)^	0.0982^(12)^	0.0000^(12)^	0.3903^(12)^	0.6875^(12)^	0.1195^(12)^	0.3903^(12)^	0.0000^(12)^	0.0983^(12)^	0.3525^(12)^	0.6875^(12)^	0.0983^(12)^	0.0000^(12)^
0.0000^(13)^	0.1365^(13)^	0.1641^(13)^	0.5130^(13)^	0.1365^(13)^	0.0000^(13)^	0.5520^(13)^	1.0631^(13)^	0.1641^(13)^	0.5520^(13)^	0.0000^(13)^	0.1371^(13)^	0.5130^(13)^	1.0631^(13)^	0.1371^(13)^	0.0000^(13)^
0.0000^(14)^	0.2129^(14)^	0.2532^(14)^	0.8339^(14)^	0.2129^(14)^	0.0000^(14)^	0.8754^(14)^	1.7028^(14)^	0.2532^(14)^	0.8754^(14)^	0.0000^(14)^	0.2148^(14)^	0.8339^(14)^	1.7028^(14)^	0.2148^(14)^	0.0000^(14)^
0.0000^(15)^	0.3208^(15)^	0.3694^(15)^	1.1300^(15)^	0.3208^(15)^	0.0000^(15)^	1.2329^(15)^	2.2316^(15)^	0.3694^(15)^	1.2329^(15)^	0.0000^(15)^	0.3080^(15)^	1.1300^(15)^	2.2316^(15)^	0.3080^(15)^	0.0000^(15)^
0.0000^(16)^	0.0742^(16)^	0.0833^(16)^	0.2592^(16)^	0.0742^(16)^	0.0000^(16)^	0.2809^(16)^	0.9053^(16)^	0.0833^(16)^	0.2809^(16)^	0.0000^(16)^	0.0698^(16)^	0.2592^(16)^	0.9053^(16)^	0.0698^(16)^	0.0000^(16)^
0.0000^(17)^	0.1125^(17)^	0.1279^(17)^	0.4197^(17)^	0.1125^(17)^	0.0000^(17)^	0.4426^(17)^	1.2809^(17)^	0.1279^(17)^	0.4426^(17)^	0.0000^(17)^	0.1086^(17)^	0.4197^(17)^	1.2809^(17)^	0.1086^(17)^	0.0000^(17)^
0.0000^(18)^	0.1889^(18)^	0.2170^(18)^	0.7406^(18)^	0.1889^(18)^	0.0000^(18)^	0.7660^(18)^	1.9206^(18)^	0.2170^(18)^	0.7660^(18)^	0.0000^(18)^	0.1863^(18)^	0.7406^(18)^	1.9206^(18)^	0.1863^(18)^	0.0000^(18)^
0.0000^(19)^	0.2968^(19)^	0.3332^(19)^	1.0367^(19)^	0.2968^(19)^	0.0000^(19)^	1.1235^(19)^	2.4494^(19)^	0.3332^(19)^	1.1235^(19)^	0.0000^(19)^	0.2795^(19)^	1.0367^(19)^	2.4494^(19)^	0.2795^(19)^	0.0000^(19)^
0.0000^(20)^	0.0383^(20)^	0.0446^(20)^	0.1605^(20)^	0.0383^(20)^	0.0000^(20)^	0.1617^(20)^	0.3756^(20)^	0.0446^(20)^	0.1617^(20)^	0.0000^(20)^	0.0388^(20)^	0.1605^(20)^	0.3756^(20)^	0.0388^(20)^	0.0000^(20)^
0.0000^(21)^	0.1147^(21)^	0.1337^(21)^	0.4814^(21)^	0.1147^(21)^	0.0000^(21)^	0.4851^(21)^	1.0153^(21)^	0.1337^(21)^	0.4851^(21)^	0.0000^(21)^	0.1165^(21)^	0.4814^(21)^	1.0153^(21)^	0.1165^(21)^	0.0000^(21)^
0.0000^(22)^	0.2226^(22)^	0.2499^(22)^	0.7775^(22)^	0.2226^(22)^	0.0000^(22)^	0.8426^(22)^	1.5441^(22)^	0.2499^(22)^	0.8426^(22)^	0.0000^(22)^	0.2097^(22)^	0.7775^(22)^	1.5441^(22)^	0.2097^(22)^	0.0000^(22)^
0.0000^(23)^	0.0764^(23)^	0.0891^(23)^	0.3209^(23)^	0.0764^(23)^	0.0000^(23)^	0.3234^(23)^	0.6397^(23)^	0.0891^(23)^	0.3234^(23)^	0.0000^(23)^	0.0777^(23)^	0.3209^(23)^	0.6397^(23)^	0.0777^(23)^	0.0000^(23)^

L.: LARGE

M.L.L.: More or less LARGE

V.L.: Very LARGE

V.V.L.: Very very LARGE

From the viewpoint of mathematical operations and the characterization of linguistic variables, the divergence/distance between the above fuzzy sets has the following requirements:
73747576

From the numerical results presented in Table 3, we see that the proposed fuzzy mean difference divergence measures (6) - (23) satisfy the requirement (73) - (76). Therefore, the proposed fuzzy mean difference divergence measures are consistent in the application with compound linguistic measures.

## Conclusion

To sum up, we present a sequence of fuzzy mean difference divergence measures. We also establish a sequence of inequalities among some of the proposed fuzzy mean difference divergence measures. An application of the proposed divergence measures in the field of pattern recognition is established. A numerical example is used to present the consistency of these divergence measures in application with compound linguistic variables. Numerical results show that the fuzzy mean difference divergence measures are much simpler with the difference of the means involved.
